# Inflammatory Mesenteric Disease and Sarcoidosis-like Reaction in a Patient with Lung Adenocarcinoma Who Received Pembrolizumab: Paraneoplastic Syndrome, Secondary to Checkpoint Inhibitor or Chance Finding?

**DOI:** 10.3390/curroncol31110540

**Published:** 2024-11-18

**Authors:** Luis Posado-Domínguez, María Escribano-Iglesias, Lorena Bellido-Hernández, Johana Gabriela León-Gil, María Asunción Gómez-Muñoz, Felipe Gómez-Caminero López, María Martín-Galache, Sandra M. Inés-Revuelta, Emilio Fonseca-Sánchez

**Affiliations:** 1 Medical Oncology Department, University Hospital of Salamanca, 37007 Salamanca, Spain; 2 Institute of Biomedical Research of Salamanca (IBSAL), 37007 Salamanca, Spain; 3 Radiodiagnosis Service, University Hospital of Salamanca, 37007 Salamanca, Spain; 4 Pathology Department, University Hospital of Salamanca, 37007 Salamanca, Spain; 5 Nuclear Medicine Service, University Hospital of Salamanca, 37007 Salamanca, Spain; 6 Internal Medicine Service, University Hospital of Salamanca, 37007 Salamanca, Spain; 7 Faculty of Medicine, University of Salamanca, 37007 Salamanca, Spain

**Keywords:** sarcoid like reaction, lung cancer, panniculitis, immunotherapy, toxicity

## Abstract

*Summary*: Anti PD1/PD-L1 agents, including pembrolizumab, have revolutionized the oncological treatment of different types of cancer, including non-small cell lung cancer. The most frequent complications associated with this type of treatment are mild and are located at the thyroid, pulmonary or hepatic level. Sarcoid like reaction and mesenteric panniculitis secondary to pembrolizumab treatment are two very rare adverse effects. We present the case of a patient with these complications. *Purpose*: the treatment of metastatic non-small cell lung cancer has undergone a major change in the last 10 years, largely due to the advent of immunotherapy. Anti PD1 agents such as pembrolizumab have increased the median survival of these patients from 13 to 26 months. Most frequent immunorelated side effects are hypothyroidism, pneumonitis or elevated liver enzymes. However, there are other adverse effects, including sarcoid-like reaction and mesenteric panniculitis, which should be known by the professionals involved in the diagnosis and treatment of this type of patient. We present the case of a 62-year-old man with a history of unresectable and non-irradiable stage IIIB epidermoid lung carcinoma with a PD-L1 expression of 30% in whom pembrolizumab was discontinued after 4 cycles due to immunorelated arthritis. One year later he consulted for severe abdominal pain. A PET-CT scan was performed, showing hilar lymphadenopathy and inflammation of abdominal mesenteric fat. A biopsy of lesions in both areas showed non-necrotizing granulomatous lymphadenitis in hilar adenopathy and patchy fibrosis of mesenteric fat. The picture was classified as sarcoidosis-like reaction and mesenteric panniculitis secondary to pembrolizumab. Anti-PD1 agents cause hyperactivation of the immune system through T-cell proliferation. Sarcoid-like reaction is a very rare complication that can mask progressive tumor disease. Awareness of immunorelated complications by oncologists, internists, and radiologists is important for an appropriate diagnostic approach and targeted test ordering.

## 1. Introduction

Inflammatory diseases of the mesentery are different aspects of the same spectrum of chronic non-neoplastic inflammation and fibrosis in this anatomical region [1]. Among these entities, an initial phase characterized by non-specific inflammation of the adipose tissue can be distinguished, known as mesenteric panniculitis (MP), which in some cases evolves into retractile mesenteritis (RM), which represents the final fibrotic phase of this process. In addition, retroperitoneal fibrosis should be considered as a distinct entity within this same subgroup of inflammatory diseases. 

MP and RM can be understood as two spectrums of the same entity, which together comprise inflammatory disease of the mesentery. Histologically, the MP phase is characterized by infiltration of mesenteric fat with lipid-laden macrophages between adipocytes, scattered lymphoid follicles, and variable degrees of fibrosis. The RM phase is dominated by an extensive degree of fibrosis [2]. 

These entities are relatively rare. In a series of autopsies, their prevalence was 1% [3], while different radiological series of abdominal computed tomography (CT) have found prevalences ranging from 0.16% to 2.5% [4,5,6]. The predominant sex differs among different series; some studies report a predominance in males [2,7], while others mention a slight predominance in females [4]. 

One-third of the cases may be asymptomatic [2]. When symptoms occur, the most common include abdominal pain, nausea, diarrhea, fever, vomiting, or fatigue [3,4,5,6,7,8]. In some cases, fibrosis can lead to intestinal obstruction. 

The diversity of underlying associated diseases indicates that inflammatory diseases of the mesentery are part of a non-specific process secondary to any intra-abdominal aggression directly and indirectly which initiates a local reaction that triggers macrophagic infiltration of fat, inflammation and fibrosis independent of the cause [5]. Among the multiple causes (Table 1), G Kuang made the first mention of immune-mediated etiology in relation to checkpoint inhibitors as a cause of mesenteritis [6].

Pembrolizumab is a humanized IgG4 monoclonal antibody that inhibits the interaction between the PD-1 protein (programmed death protein 1) present on T cells of the immune system with its ligand (PD-L1) present on tumor cells resulting in the activation of the immune system to attack tumor cells [9]. Although it has revolutionized the treatment of non-small cell lung cancer (NSCLC) by markedly increasing median survival, this treatment can cause adverse effects as a result of excessive activation or dysregulation of the immune system, the most common of which include skin eruptions, colitis, pneumonitis, hepatitis, and thyroid alterations, as well as other rarer complications also described, such as encephalitis [10,11,12].

Immune-mediated panniculitis is a rare complication, with fewer than 12 cases described in the literature, most of them in patients with melanoma in whom granulomatous or lobar reactions are present in the subcutaneous fat at the site of injury [13,14].

Sarcoidosis is a multisystem granulomatous disease characterized by the accumulation of T lymphocytes, activated macrophages, and non-caseating granulomas in response to unknown antigens. Diagnosis is based on a typical clinical and radiological presentation together with histologically confirmed non-caseating granulomas and the exclusion of alternative diseases, including tuberculosis and cancer-associated sarcoid reaction [1,15]. Antitumor agents such as immunotherapy or some traditional chemotherapeutics such as cisplatin can induce through an immune response the development of sarcoid reactions in different known anatomical areas, known as sarcoidosis-like reaction (SLRs) [16]. The development of these reactions is related to the modulation of macrophages and T cells, mainly CD4+ T lymphocytes, which play a crucial role in the formation of non-caseating granulomas. Immune checkpoint inhibitors, such as pembrolizumab, by blocking the interaction between the PD-1 receptor of T lymphocytes and its ligand PD-L1 on tumor cells, eliminate a key mechanism of immune suppression, triggering an exaggerated activation of PD-1+ T lymphocytes. This uncontrolled activation of T lymphocytes results in the release of various proinflammatory cytokines, such as interleukin-2 (IL-2), which in turn stimulates the proliferation and activation of more T lymphocytes.

Cytokines released by activated T lymphocytes attract and activate macrophages, which are the cells responsible for producing and secreting tumor necrosis factor-alpha (TNF-α), one of the main molecules involved in the inflammatory response and granuloma formation. TNF-α induces the accumulation and differentiation of macrophages into epithelioid cells and multinucleated giant cells, promoting the formation of granulomas that are characteristic of sarcoidosis and sarcoidosis-like reactions.

In this process, both T lymphocytes and macrophages play essential roles by collaborating to maintain the granulomatous inflammatory response.

Thus, the development of sarcoidosis-like reactions is mediated by a dysregulated activation of T lymphocytes, which triggers the release of IL-2 and the activation of macrophages, which in turn produce large amounts of TNF-α. This inflammatory cycle perpetuates the formation of granulomas in multiple organs.

Here we describe the case of a patient with adenocarcinoma of the lung who presented with mesenteric panniculitis and a sarcoidosis-like reaction in probable relation to pembrolizumab.

**Table 1:** Proposed etiologic causes of inflammatory mesenteric disease. Hypothesis explaining this cause. References both for and against it.

Etiology	Hypothesis	References
Abdominal surgery Abdominal trauma	- Problems in connective tissue repair due to an exacerbated inflammatory response following surgical or physical trauma.	Silvia Carbonell et al. J Ruiz-Tovar et al. [5,7]
Paraneoplastic syndrome	- Abnormal inflammatory immune response originating from tumor cells mediated by cytokines or products of tumor metabolism. - Systemic tumor inflammation affecting mesenteric tissue.	For - A.J. Cross et al. - F.Scheer et al. - Okino Y. Kiyosue H et al. Against - P. Buchwald et al. - O. Gogeebakan et al. [2,4,13,14,15]
Toxicity to immunotherapy	- Hyperactivation of the immune system through T cells.	G kuang et al. [6]
Autoimmune conditions	- Chronic inflammation including changes in mesenteric adipose tissue. - Changes in the immune system causing activation of immune cells against some antigens of the mesenteric adipose panniculus.	Silvia Carbonell et al. J Ruiz-Tovar et al. [5,7]
Ischemic pictures	- Interruption of blood flow to the mesentery. - Tissue damage by hypoxia of adipose tissue with subsequent repair and fibrosis as part of the healing process.	WW Ogden et al. A De la Peña et al. [17,18]
Ureteral lithiasis	- Inflammation of the mesentery due to proximity to the ureter. - Proximal mesenteric fat ischemia - Immune reaction, activation of the immune system in response to the stone.	Adeleh Dadkhah et al. Serra Ozbal et al. [19,20]
Previous infections (bacterial or viral infections in the abdomen, appendicitis, diverticulitis).	- Inflammatory reaction in the mesentery that evolves into inflammation of the adipose panniculus. - Fibrosis and necrosis of fat tissue in response to inflammation.	Silvia Carbonell et al. G Ege et al. [5,21]
Inflammatory bowel disease		J Ruiz-Tovar et al. [7]
Metabolic factors (Obesity, diabetes mellitus, metabolic syndrome, etc).	- Chronic proinflammatory state - Imbalance in the release of proinflammatory cytokines by adipose tissue. - Oxidative stress - Hypercholesterolemia	Serra Ozbal et al. Hagai Scheweistein et al. [20,22]
Idiopathic		Silvia Carbonell et al. J Ruiz-Tovar et al. [5,7]

## 2. Clinical Case

We present the case of a male between 60 and 70 years of age with no history of hypertension, diabetes mellitus, dyslipidemia, or cardiovascular disease. Body mass index of 23, LDL cholesterol in normal range, no history of urolithiasis or previous abdominal surgery. Independent for basic and instrumental activities of daily living, active worker in the hospitality industry. Ex-smoker of 1 pack of cigarettes per day, non-drinker.

**Figure 1:**
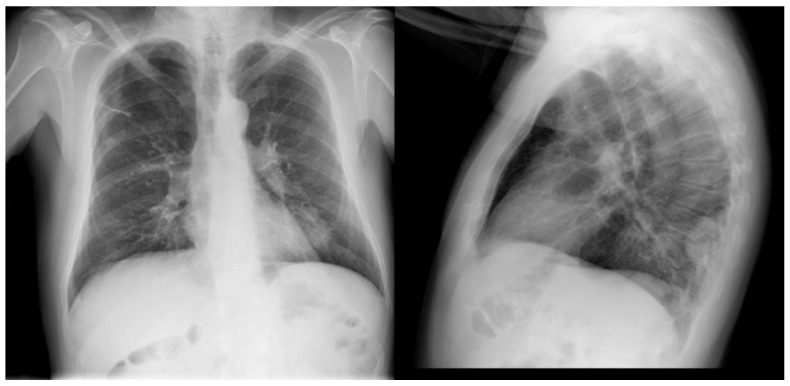
Chest X-ray in posteroanterior and lateral projection. In the left lower lobe there is a pulmonary mass of approximately 5 cm, suggestive of tumor origin, to be completed with scheduled CT study. Diminution of the vascular network with increased lucidity of the pulmonary parenchyma in both upper lobes, predominantly right, probably related to emphysematous changes. Laminar atelectasis in the LLB. Bilateral hilar prominence.

In January 2021, he consulted for general malaise, myalgias, and fever and was diagnosed with SARS-CoV-2 infection after a positive antigen test. The chest X-ray showed a suspicious lung mass (Figure 1), so it was decided to perform a thoracoabdominal-pelvic CT scan which revealed a lung carcinoma in the left lower lobe (LLB) with bilateral hilar adenopathies, radiological staging T2a N3 M0 Stage IIIB. Biopsy confirmed a lung adenocarcinoma with PD-L1 expression of 30% and mutation in KRAS G13X, negative for EGFR, ALK, and ROS1.

Initially, chemo-radiotherapy (QT-RT) was considered, but radiotherapy was discouraged due to the extent of the tumor field. The patient started treatment with cisplatin-pemetrexed-pembrolizumab in March 2021. In the second cycle, cisplatin was replaced by carboplatin due to alterations in renal function. After 4 cycles of treatment, the patient presented a partial response and continued with maintenance pemetrexed-pembrolizumab.

In October 2021, he attended the emergency department for severe abdominal pain, joint pain in both ankles, and edema with increased local temperature. Laboratory tests showed mild leukocytosis, elevated erythrocyte sedimentation rate (ESR), and C-reactive protein (CRP), which led to the diagnosis of immune-mediated arthritis secondary to treatment with PD-1/PD-L1 inhibitors. He started treatment with prednisone 10 mg/day and a re-evaluation PET-CT scan was requested.

PET-CT showed a persistence of hypermetabolic mass in the left lower lobe (increased SUV) with normalization of hilar adenopathy and the appearance of pseudonodular formations in the mesenteric fat suggestive of mesenteric panniculitis. It was concluded that mesenteric panniculitis could be related to the use of pembrolizumab. The patient was continued on prednisone and first step analgesia with good control of abdominal pain and resolution of arthritis.

In November 2021, after 8 sessions of stereotactic radiotherapy (SBRT) on the lung lesion, the re-evaluation CT scan showed a decrease in the size of the tumor. It was decided to discontinue pemetrexed-pembrolizumab and maintain observation.

Disease control was maintained until the end of August 2022, when a new CT and PET-CT scan was performed, showing thoracic lymph node progression, right lower cervical adenopathy and hiliomediastinitis. Signs of residual mesenteric panniculitis were observed with practical metabolic normalization of the FDG uptake foci at this level that were visualized in the previous PET scan of October 2021.

**Figure 2:**
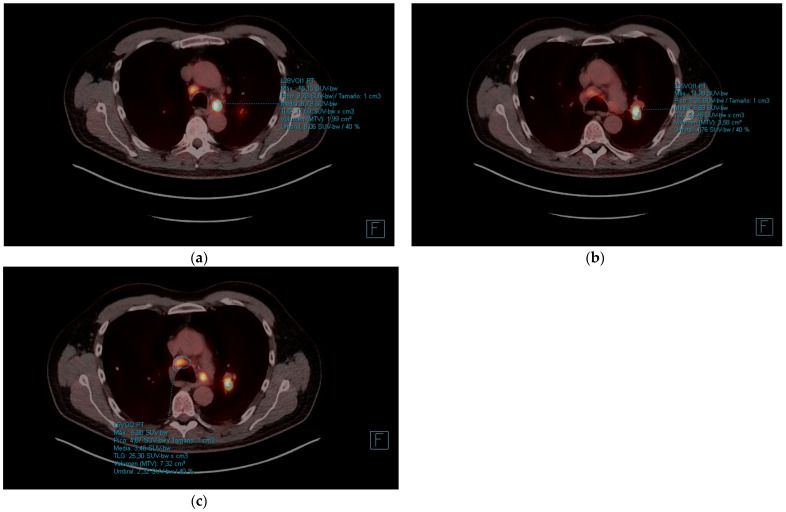
Standard PET-CT scan. Multiple hypermetabolic adenopathies, the most representative being left paratracheal (**a**) (SUVmax 15), left hilar (**b**) (SUVmax 11.9), and subcarinal (**c**) (SUVmax 5.8). Biopsy showed a non-necrotizing lymphogranulomatous reaction.

**Figure 3:**
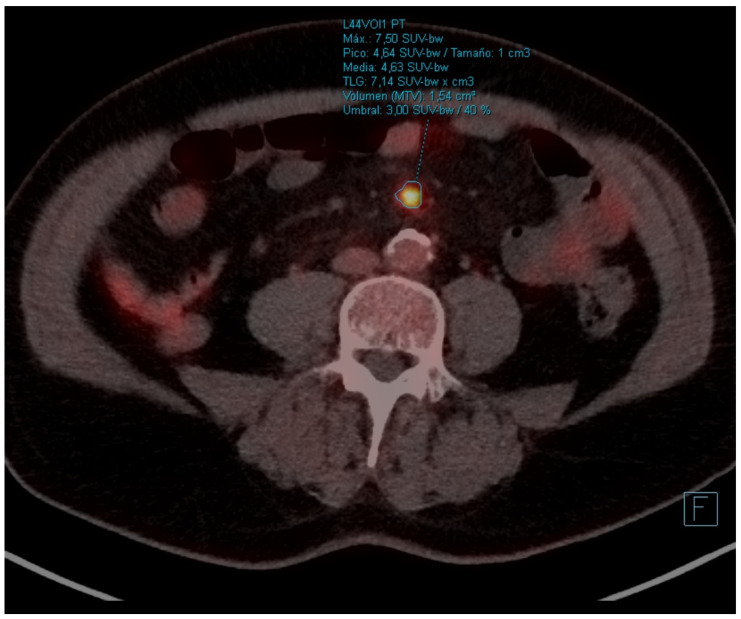
Standard PET-CT. Signs of mesenteric panniculitis, visualizing multiple pseudonodular formations several of them with FDG uptake up to SUVmax 7.50.

In early October 2022, he started treatment according to the docetaxel-nintedanib regimen. The docetaxel dose was adjusted due to gastrointestinal toxicity (diarrhea, nausea). In January 2023, CT scan was performed with stabilization of the disease. Between March and April 2023, he consulted the emergency department 4 times for severe abdominal pain, nausea, and diarrhea. Given the history of mesenteric panniculitis, it was decided to perform a PET-CT scan with the finding of hypermetabolic persistence of adenopathies in the right cervical chain, lower paratracheal and right pulmonary hilum with dissociated response, some have progressed and others have improved (Figure 2a—left paratracheal, Figure 2b—left hiliar and Figure 2c—subcarinal), and signs of mesenteric panniculitis, visualizing an increase in pseudonodular formations, several with FDG uptake up to SUVmax 7.50 not present in the previous study (Figure 3).

In view of the reactivation of the mesenteric panniculitis, prednisone was restarted at a dose of 10 mg/day and due to poor analgesic control, oxycodone/naloxone was started.

Given the dissociated lymph node response, it was decided to perform a bronchoscopy with biopsies with findings of non-necrotizing granulomatous reaction in adenopathic regions 4R and 7. The picture was classified as sarcoidosis-like reaction in possible relation with having received immunotherapy. Since there was no evidence of disease, docetaxel-nintedanib was suspended and observation was decided.

The patient continued with episodes of diarrhea and pain, so in July 2023 a new abdominal CT scan was performed showing hilar lymph node enlargement, and an exploratory laparoscopy with biopsy of the mesentery showed findings of extensive mesenteric fibrosis.

**Figure 4:**
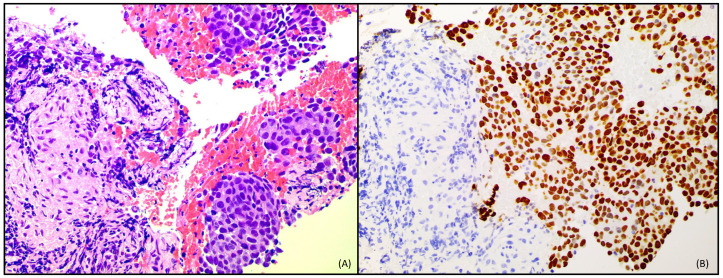
(**A**) Hematoxylin-eosin staining: epithelioid histiocytes forming a non-necrotizing granuloma, with a group of tumor cells, positive for TTF1 immunohistochemistry. (**B**) TTF1 immunohistochemistry. 40× magnification.

Given the growth of the hilar adenopathies, a new bronchoscopy with biopsy was performed, observing persistent non-necrotizing granulomatous reaction in region 4R and metastasis of adenocarcinoma on non-necrotizing granulomatous reaction in region 7 (Figure 4). The most important complementary explorations and their results can be seen in Table 2. Docetaxel-Nintedanib was reinitiated and maintained until January 2024 (4 cycles) when lymph node progression was observed.

**Figure 5:**
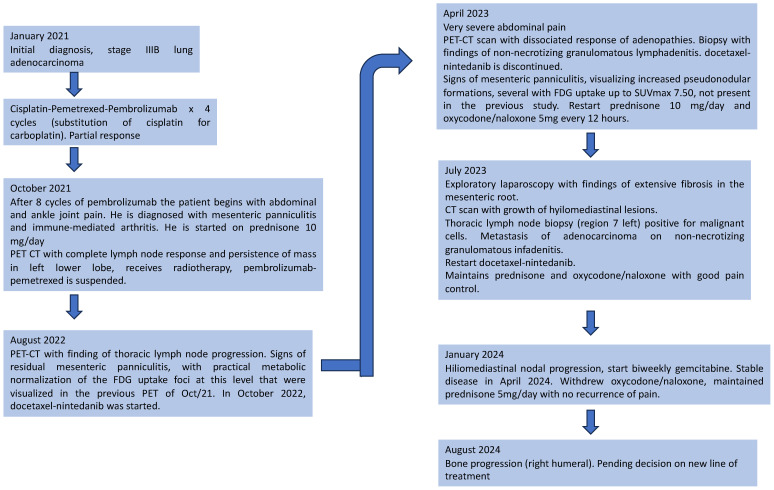
Timeline of events that occurred to the patient between January 2021 and August 2024. The oncological diagnosis, the diagnosis of immune-mediated arthritis and mesenteric panniculitis, and the finding of a granulomatous reaction in a hilar biopsy are reported.

In January 2024, he started third line of treatment with biweekly gemcitabine, stable disease in April 2024. In July 2024, bone progression of the disease was observed and a new line of treatment is currently pending. A schematic summary of the events over time can be seen in Figure 5.

## 3. Discussion

The advent of immunotherapy, particularly immune checkpoint inhibitors such as pembrolizumab, has revolutionized the treatment of various cancers, including melanoma, lung cancer, and more recently, urothelial carcinoma. Immune-mediated reactions have become increasingly significant in routine clinical practice, as demonstrated by numerous reviews on diagnostic processes and treatment algorithms for common conditions such as thyroiditis, hepatitis, and colitis. However, rare complications like mesenteric panniculitis or sarcoidosis-like reactions have been infrequently reported in the literature, and their implications and management remain unclear, highlighting the importance of documenting and reporting such cases.

With respect to immune-mediated panniculitis, one of the few cases described in the literature was reported by G kuang et al. [6] in relation to a case of mesenteritis secondary to pembrolizumab. In the case of our patient, the diagnosis was made when he initially consulted in October 2021 due to intense abdominal pain, as well as CRP and ESR elevation together with mild leukocytosis and pseudonodular formations in the mesenteric fat with PET-CT uptake (Figure 5). The onset of clinical manifestations compatible with panniculitis together with arthralgia and periarticular inflammation of both ankles suggested at that time an immunorelated etiology coinciding with the administration of pembrolizumab in recent months. Although the exact pathogenesis remains unclear, it is plausible that pembrolizumab, by modifying the immune balance, contributed to the development of panniculitis through complex mechanisms of immune system activation. The good response to prednisone supported this diagnosis. However, the patient presented recurrent episodes of abdominal pain associated with recurrence of mesenteric panniculitis more than a year later, which required symptomatic management with third step analgesia and implied the performance of a mesenteric biopsy for confirmation; it is worth mentioning that despite the recurrence, the patient did not present other serious complications such as intestinal obstruction.

When in April 2023, after observing a dissociated response of the hilar adenopathies, it was decided to biopsy the hilar adenopathies, a sarcoidosis-like reaction was observed. At that time, thinking retrospectively, it was hypothesized that the periarticular inflammation of both ankles could have been Lofgren’s syndrome, an acute form of sarcoidosis with a favorable prognosis [23]. In that case, after observing two years later the lymphogranulomatous reaction suggestive of sarcoid reaction in thoracic lymph node regions, it is worth hypothesizing whether immunotherapy played a role in inducing a sarcoidosis process in two locations or reactivated a latent sarcoidosis. Although induced sarcoidosis is rare, cases such as this one reinforce the need to be alert to the appearance of granulomatous manifestations in patients under immunotherapy.

A crucial aspect of the management of these sarcoidosis-like reactions is the need to perform biopsies to differentiate between an immune-mediated reaction and a progression or recurrence of the primary malignancy [24]. In our case, the initial biopsy showed granulomatous lesions typical of sarcoidosis but a subsequent biopsy revealed adenocarcinoma metastases on sarcoid lesions, which underlines the importance of periodically re-evaluating this type of lesions to avoid misdiagnosis.

## 4. Conclusions

Despite the limited literature available on mesenteric panniculitis secondary to pembrolizumab, it is suggested that immunotherapy may have played a key role in activating or exacerbating an inflammatory response in mesenteric adipose tissue. However, in the absence of further studies and more documented cases, it is difficult to draw definitive conclusions about the exact pathophysiology of this complication.

When mesenteric panniculitis is suspected, a differential diagnosis including other pathologies such as mesothelioma, amyloidosis, liposarcoma or peritoneal carcinomatosis is essential, as some of these conditions may present similar clinical and radiological manifestations. Furthermore, in some cases, mesenteritis may coexist with lymphomas, highlighting the need for a comprehensive diagnostic approach. In the coming years, with the accumulation of more case reports, we will be able to determine whether mesenteric panniculitis is a rare but significant adverse effect of immunotherapy, which will allow us to establish clear guidelines for its management.

Regarding sarcoidosis-like reactions, it is important for multidisciplinary teams assessing cancer patients to be aware of the existence of this rare adverse effect. The likely increase in the number of cases in the coming years due to the increasing use of immunotherapy will allow radiologists and nuclear medicine physicians to better evaluate imaging tests in the differential diagnosis of this reaction. However, we believe that confirmatory biopsy is essential as a sarcoid reaction may mask tumor progression.

The next few years will be crucial to deepen the management of this complication, allowing the establishment of clear guidelines on whether it is necessary to suspend or continue immunotherapy in these patients, optimizing treatment strategies.

## Figures and Tables

**Table 2:** PET-CT scans and biopsies performed on the patient between March 2021 and April 2023.

Test	Date	Moment	Findings
PET-CT	March 2021	Initial diagnosis, staging	- Hilar adenopathy compatible with malignancy (SUVmax 20.7). - Hypermetabolic mass (SUVmax 18.3) in the left lower lobe measuring 3.2 × 5.1 cm compatible with malignancy. - Calcified granuloma in right upper lobe. - Abdomen without foci of pathological uptake.
PET-CT	October 2021	Re-evaluation	- Hypermetabolic mass (SUVmax 24.72) in left lower lobe of 4.2 × 3.5 cm. - Complete response of the hilar adenopathies, without pathologic uptake. - Focal increase of FDG in pseudonodular formations in mesenteric fat, up to SUVmax 12.48, in relation to mesenteric panniculitis.
PET-CT	October 2022	Re-evaluation	- Morphometabolic progression of pathologic adenopathies observed on staging PET in March 2021 that had normalized in October 2021, SUVmax 19.5. - Complete morphometabolic response of primary left lower lobe neoplasm, currently 1.8 cm and SUVmax of 2.6. - Signs of mesenteric panniculitis, with practical metabolic normalization of the FDG uptake foci at this level that were visualized in the previous PET scan of Oct/21.
PET-CT	April 2023	Severe abdominal pain that had consulted 4 times in the emergency room	- Hypermetabolic persistence of adenopathies in the right cervical chain, lower paratracheal and right pulmonary hilum with dissociated response, some have progressed and others have improved. - Signs of mesenteric panniculitis, visualizing increased pseudonodular formations, several with FDG uptake up to SUVmax 7.50, not present in the previous study.
Biopsy of hilar adenopathy	April 2023	Sarcoid reaction vs. tumor progression	- Transbronchial FNA of region 4R: non-necrotizing granulomatous reaction. - Transbronchial FNA of region 7: non-necrotizing granulomatous reaction.
Mesentery biopsy in exploratory laparoscopy	July 2023	Evaluation of mesenteric panniculitis. Rule out other causes of abdominal pain.	- Biopsy of transverse mesocolon: scanty patchy fibrosis. - Biopsy of greater omentum: scanty patchy fibrosis. - Mesenteric root biopsy: extensive fibrosis.
Biopsy of hilar adenopathy	July 2023	Sarcoid reaction vs. tumor progression	- Transbronchial FNA of region 4R: non-necrotizing granulomatous reaction. - Transbronchial FNA of region 7: adenocarcinoma metastasis on non-necrotizing granulomatous reaction, TTF1+, p40-.

## Data Availability

The clinical data collected for this research are available in patient clinical databases managed by the Medical Oncology Department of the University Hospital of Salamanca. These data, in anonymized form, can be used by external researchers for future investigations.
